# Vitamin C: Intravenous Use by Complementary and Alternative Medicine Practitioners and Adverse Effects

**DOI:** 10.1371/journal.pone.0011414

**Published:** 2010-07-07

**Authors:** Sebastian J. Padayatty, Andrew Y. Sun, Qi Chen, Michael Graham Espey, Jeanne Drisko, Mark Levine

**Affiliations:** 1 Molecular and Clinical Nutrition Section, National Institute of Diabetes and Digestive and Kidney Diseases, National Institutes of Health, Bethesda, Maryland, United States of America; 2 Program in Integrative Medicine, University of Kansas Medical Center, Kansas City, Kansas, United States of America; University of Michigan, Canada

## Abstract

**Background:**

Anecdotal information and case reports suggest that intravenously administered vitamin C is used by Complementary and Alternate Medicine (CAM) practitioners. The scale of such use in the U.S. and associated side effects are unknown.

**Methods and Findings:**

We surveyed attendees at annual CAM Conferences in 2006 and 2008, and determined sales of intravenous vitamin C by major U.S. manufacturers/distributors. We also queried practitioners for side effects, compiled published cases, and analyzed FDA's Adverse Events Database. Of 199 survey respondents (out of 550), 172 practitioners administered IV vitamin C to 11,233 patients in 2006 and 8876 patients in 2008. Average dose was 28 grams every 4 days, with 22 total treatments per patient. Estimated yearly doses used (as 25g/50ml vials) were 318,539 in 2006 and 354,647 in 2008. Manufacturers' yearly sales were 750,000 and 855,000 vials, respectively. Common reasons for treatment included infection, cancer, and fatigue. Of 9,328 patients for whom data is available, 101 had side effects, mostly minor, including lethargy/fatigue in 59 patients, change in mental status in 21 patients and vein irritation/phlebitis in 6 patients. Publications documented serious adverse events, including 2 deaths in patients known to be at risk for IV vitamin C. Due to confounding causes, the FDA Adverse Events Database was uninformative. Total numbers of patients treated in the US with high dose vitamin C cannot be accurately estimated from this study.

**Conclusions:**

High dose IV vitamin C is in unexpectedly wide use by CAM practitioners. Other than the known complications of IV vitamin C in those with renal impairment or glucose 6 phosphate dehydrogenase deficiency, high dose intravenous vitamin C appears to be remarkably safe. Physicians should inquire about IV vitamin C use in patients with cancer, chronic, untreatable, or intractable conditions and be observant of unexpected harm, drug interactions, or benefit.

## Introduction

Among the most enduring of alternative medical treatments, vitamin C (ascorbic acid, ascorbate) is also one of the most popular. In 2007, it was the most widely sold single vitamin, with sales of 884 million dollars in the US1 [1]. Independent of its use to treat the deficiency disease scurvy, vitamin C has been used by non-mainstream physicians orally and parenterally for more than 60 years as a therapeutic agent [2]–[7]. Oral vitamin C is widely used by the public to prevent or treat infections, especially the common cold [8]. In one of its more controversial applications, gram doses of vitamin C were promoted by the two-time Nobel Laureate Linus Pauling as a cancer treatment agent [9], [10]. Anecdotal evidence led us to posit that intravenous (IV) vitamin C is still used by Complementary and Alternative Medicine (CAM) practitioners to treat diverse conditions including infections, autoimmune diseases, cancer and illnesses of uncertain origin [11]–[13].

Despite its purported popularity, the extent of use of IV vitamin C is unknown. Its use in CAM has not been well publicized by practitioners and their patients, and is likely to be unrecognized by mainstream physicians. Benefits if any and especially side effects of such use may be unreported or under-reported. It is useful to know if high dose IV vitamin C therapy is widely used, and if so how and for what, so that conventional physicians can improve patient care by identifying any ill effects or drug interactions, and reporting benefit if any.

New knowledge has elucidated possible mechanisms of action of IV vitamin C and for the first time made therapeutic effects biologically plausible [14]. It is now known that IV but not oral administration of vitamin C produces pharmacologic plasma concentrations of the vitamin [15], [16]. Past studies used oral and/or IV routes inconsistently, making such studies, in retrospect, flawed and difficult to interpret [17]. Recent *in vitro* experiments indicated that vitamin C only in pharmacologic concentrations killed cancer cells but not normal cells, and that the mechanism was via hydrogen peroxide formation [18]. *In vivo* animal data indicated that hydrogen peroxide was produced selectively in extracellular fluid around normal and tumor tissues by pharmacologic vitamin C concentrations [19], [20]. At these concentrations, vitamin C slowed tumor growth [20], [21]. Pharmacologic vitamin C concentrations produced in animals by parenteral administration were reproduced in patients in a recent phase I clinical trial [16].

Because of the new interest in IV vitamin C, coupled to need to characterize use and uncover side effects, we surveyed CAM practitioners anonymously. We also searched for side effects of IV vitamin C administration in the published medical literature and in the Food and Drug Administration (FDA) adverse events database, and estimated sales volumes of IV vitamin C preparations.

Our study obtained quantitative information that substantiated previous anecdotal reports. Despite unexpected wide use, we found side effects of vitamin C were surprisingly few when patients were properly screened. The findings in this paper will alert conventional practitioners about unrecognized wide use of IV vitamin C, will remind them to query patients about such use, and may help to uncover either unexpected adverse events or benefit and spur further research in this area.

## Methods

### Survey Methods

The study was reviewed by the Human Subjects Committee/Institutional Review Board at the University of Kansas Medical Center. The survey was categorized as an exempt study. It contained no personal identifiers; therefore informed consent was not necessary under exempt status and was not obtained. Survey forms were distributed to practitioners attending a conference on CAM in 2006 and 2008. Participants were requested to return completed survey forms before the end of each conference. Participants were asked whether they used high dose IV vitamin C during the preceding 12 months, and if so, to detail its use by answering specific questions in the survey form (see Survey S1 for the survey form used). It was not possible to identify the respondent from the survey form or survey data. The same form was used in both years, with an additional line on the 2008 form inquiring whether the respondent had also responded in 2006 (See Survey S1).

### Vitamin C doses sold

The major manufacturers/importers in the US of vitamin C preparations that can be administered IV were contacted by telephone. Data on annual sales of vitamin C in the US were obtained with the understanding that the names and sales figures of individual companies would not be linked nor made public.

### Side effects of high dose IV vitamin C

We searched the Adverse Events Reporting System, a database of drug side effects maintained by the Food and Drug Administration (FDA). Data from 20 consecutive quarters available from 2004–2008 were queried. We also searched for side effects in publications on therapeutic use of high dose IV vitamin C. We searched Medline, Web of Science (ISI Thompson) and Scopus databases for papers in English that reported IV vitamin C administration in humans. Several different search terms and possible variants of each search term were used to capture the maximum number of papers. Papers reporting oral vitamin C treatment only, or those using IV doses of 1g or less, were excluded. Because vitamin C is sometimes administered IV in patients undergoing hemodialysis, there are many published studies in this area. A separate search was conducted for papers reporting these to ensure that these were excluded from our analysis. IV administered vitamin C is also used to study the acute effects of antioxidants or of vitamin C itself on metabolism and physiology, particularly on cardiovascular and endothelial reactivity. This does not constitute a therapeutic use of vitamin C. Hence publications reporting these were identified and removed from the search results. Separately, we searched the same databases for specific reports of side effects of IV administered vitamin C and followed up references and cited papers. From papers so collected, we manually eliminated duplicate citations and those that did not meet the above criteria. From the remaining reports, adverse reactions attributable to IV administered vitamin C were noted.

## Results

### Survey response

We distributed 300 survey forms in 2006 to attendees at their annual CAM conference. 106 forms were returned, a response rate of 35%. In 2008, 250 survey forms were distributed and 93 completed forms were returned, a response rate of 37%. Of the 2008 respondents, 22 (24%) had previously responded in 2006. An unknown number of conference attendees were not practitioners but spouses, researchers or industry representatives who did not return survey forms, reducing response rates.

### Vitamin C usage

**Figure 1:**
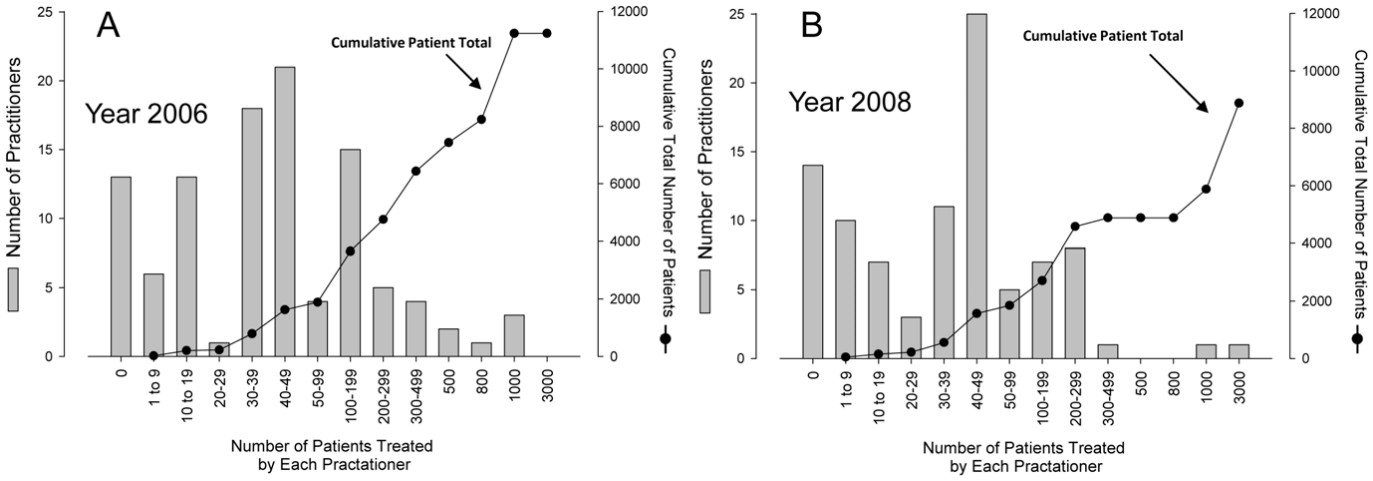
Cumulative total and the distribution of patients treated among the survey respondents. Practitioners were grouped according to the number of patients whom they treated with IV vitamin C in the preceding twelve months. For ease of display, number of patients were divided into arbitrary ranges which are shown on the X axis. The number of practitioners who treated the specified number of patients are shown on the Y axis. Patients were unevenly distributed among survey respondents. In 2006, out of 106 practitioners who responded to the survey, 13 did not use high dose IV vitamin C, 30 respondents treated more than 100 patients each, and 3 treated 1000 or more patients (Fig 1A). For 2008, the corresponding numbers were 93, 14, 18 and 2 respectively (Fig 1B). Cumulative total of patients treated by all practitioners are shown as line diagrams (scaled to Y axis on right).

Of 199 total respondents for 2006 and 2008: 172 practitioners administered vitamin C; 27 did not use IV Vitamin C; 48 practitioners treated more than 100 patients each per year; and 5 treated more than 1000 patients each per year (Figure 1A, B). 11,233 patients received IV vitamin C in 2006 and 8876 in 2008 (figure 1A, B). On average, each patient received 22 treatments (Table 1). Treatments occurred at a mean of once every 4 days, each at a mean dose of 28 grams (Table 1). Doses used were as low as 1gram or as high as 200grams, with a similar wide range for each of the parameters queried. Based on dosing vials of 25g/50ml, estimated total yearly dosing vials administered were 318,539 in 2006 and 354,647 in 2008. Estimated total number of dosing vials sold was independently obtained from the major manufacturers of vitamin C in the U.S. Total dosing vials of IV vitamin C sold in the United States were approximately 750,000 in 2006 and 855,000 in 2008 (Table 1).

**Table 1:** Details of high dose IV vitamin C use by survey respondents for the years 2006 and 2008.

	2006			2008		
	Mean	Median	Range	Mean	Median	Range
**Dose (g/treatment)**	28	31	1–200	28	50	1–200
**Number of treatments per patient**	19	16	1–80	24	16	1–80
**Number treated by one practitioner**	121	40	1–1150	112	40	1–3000
**Duration of treatments (min)**	105	90	2–1440	81	90	1–900
**Frequency of treatments (once every so many days)**	4	3.5	1–7	4	2	1–7
**Lowest dose (g/treatment)**	12	9	1–60	17	15	1–75
**Highest dose (g/treatment)**	79	75	5–200	87	95	20–200
**Infusion rate (g/min)**	0.89	0.5	0.03–25	0.525	0.5	0.028–2.5
**Total number of vials of vitamin C used (25g/50ml) (Calculated from survey data)**	318,539			354,647		
**Total number of vials of vitamin C sold by companies in the US (25g/50 ml)**	750,000			855,000		

Vitamin C is supplied in 50 ml bottles containing 25grams. Estimated total number of doses (bottles) used each year was calculated as the cumulative sum of each practitioners' number of patients×that practitioners' average dose in bottles×that practitioner's average number of doses per patient.

**Figure 2:**
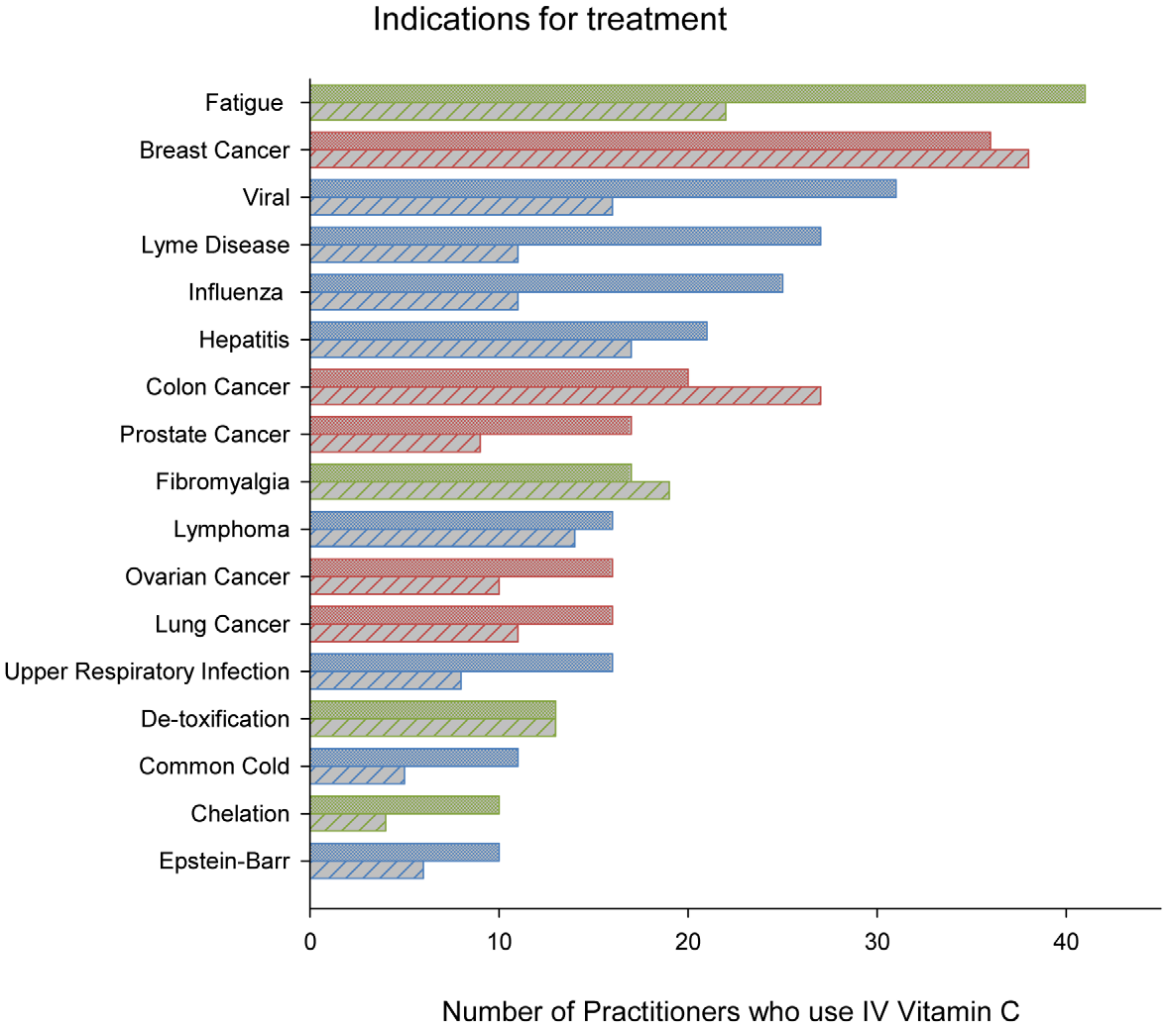
Number of practitioners who used intravenous vitamin C for various conditions. X axis shows the number of practitioners who used intravenous vitamin C to treat each of the conditions listed on the Y axis. Blue bars denote infections, red bars denote cancers, and green bars denote other indications. Data for 2006 (solid bars) and 2008 (hatched bars) show that intravenous vitamin C was most often used to treat infections and cancer. Indications for which less than four practitioners used high dose intravenous vitamin C are listed in Table S1.

Seventy seven percent of respondents reported the numbers of patients treated for broad indications, labeled as infection (44%), cancer (19%) or other conditions (37%) (Table 2). Numbers of practitioners who listed specific indications for treatment are shown in figure 2. Practitioners listed fatigue as the most common specific single indication for treatment in 2006, and breast cancer in 2008. There were a large number of indications for which less than four practitioners used high dose IV vitamin C (Table S1).

**Table 2:** Indications for treatment with high dose IV vitamin C.

Year		2006	2008
***Total number of patients treated***		*11233*	*8876*
***Number of patients with data available***		*9481*	*5928*
Number of Patients with	**Infection**	4587	2264
	**Cancer**	1379	1509
	**Other Conditions**	3515	2155

Some respondents did not list the number of patients treated for each of the conditions for which they used intravenous vitamin C treatment. Therefore, the data do not provide indications for treatment for all patients who received IV vitamin C.

### Adverse effects

Adverse events reported by survey respondents were minor (Table 3). No side effects were reported for 9227 patients while 59 were reported to have lethargy or fatigue. A single practitioner listed change in mental status in 10% of his patients (20 patients) but provided no details. One patient with pre existing renal impairment and cancer metastases to kidneys was reported to have developed unconfirmed renal failure. Some practitioners reported side effects without reporting patient numbers. The most common of these side effects were lethargy or fatigue (reported by 27 practitioners), vein irritation (by 9 practitioners), and nausea and vomiting (by 9 practitioners). Other reported side effects are listed in table 3. Less commonly reported adverse effects are listed in Table S2.

**Table 3:** Adverse events reported with IV vitamin C use in the survey for the years 2006 and 2008.

	Number of Patients	
Complication	2006	2008
**None Described**	5349	3878
**Lethargy/Fatigue**	10	49
**Local Vein Irritation**	3	-
**Phlebitis**	3	-
**Kidney Stone (oxalate)**	-	1
**Kidney Stone (urate)**	-	1
**Kidney Stone (unspecified)**	2	-
**Hemolysis**	2	-
**Elevated Blood Glucose**	2	-
**Muscle Cramps**	1	-
**Headache**	1	-
**Change in Mental Status**	1	20
**Nausea/Vomiting**	1	-
**Flu Like Syndrome**	1	-
**Renal Failure**	-	1*
**Syncope**	-	1
**Pain at Tumor**	-	1
**No Data**	5857	4924

Data included in the table represent only those practitioners who reported exact patient numbers.

*Described as “not confirmed (possible). Patient had partial renal failure and cancer metastases to kidneys.”

Data on practitioners who reported adverse events but did not report the number of patients affected are detailed below as: side effect (with the number of practitioners who reported each side effect in parenthesis).

*For the year 2006:* lethargy/fatigue (9), local vein irritation (3), nausea/vomiting (2), hypoglycemia (2), allergy (2), phlebitis (1), cellulitis (1), hematuria (1), dry mouth (1), Herxheimer reaction (1), localized thrombosis (1), and syncope (1).

*For the year 2008*: lethargy/fatigue (9), nausea/vomiting (6), local vein irritation (4), headache (3), phlebitis (3), heartburn (1), dizziness (1), venosclerosis (1), mild palpitation (1), and cold (1), dizziness (1), “initiation of mem occasionally” (1), and other (1).

Data obtained from the FDA Adverse Events Reporting System database for 20 consecutive quarters indicated that 77 patients treated with 0.2–1.0 gram doses of IV vitamin C had reported adverse events (Table 4). However, all patients either had serious or life-threatening systemic illnesses, and/or were receiving many potentially toxic drugs (i.e. cancer therapeutics) in addition to IV vitamin C (for details, see Table S3). Some individual patients appear to have been reported multiple times (see Table S3). In comparison to CAM practitioners, the dose of vitamin C administered was very low (1 gram or less). In no case could we exclude multiple confounding factors as the cause of the reported adverse effects (Table 4). Whether vitamin C caused or contributed to these side effects cannot be determined from the available data.

**Table 4:** Adverse effects reported to the Food and Drug Administration (FDA) in patients treated with IV vitamin C.

Year	Number of Cases reported	Dose Range (g/day)	Can confounders be eliminated?
2004	15	0.5–1	No
2005	7	0.25–1	No
2006	11	0.2–1	No
2007	11	Not Given	No
2008	33	0.5–1	No

When the vitamin C dose was provided as ml, the dose was converted to mg on the basis that vitamin C is supplied as 0.5gram/ml solution. Some practitioners did not mention the dose of vitamin C used. The format of the FDA adverse events database did not permit identification of specific patients, so that the same patient may have been reported multiple times in the same quarter or in several quarters, inflating the number of patients with adverse events.

Through searching published literature (see methods for details), 187 papers were found on the use of high dose IV vitamin C, including papers that reported side effects. There were three cases of renal failure, all in patients with pre existing renal impairment [22], [23], [24]. Two patients with glucose 6 phosphate dehydrogenase deficiency developed hemolysis [25], [26] (Table 5).

**Table 5:** Adverse effects of vitamin C reported in the literature.

#	Type of Side Effect	Patient Details	Vitamin C Dose	Clinical Details		Outcome (and reference)
				Pre Vitamin C Treatment	Post Vitamin C Treatment	
1	Acute Renal Failure	70 M	2.5g IV×1 dose	Creatinine 5.0	Flank pain, hematuria. Creatinine 10.	Permanent renal failure(24)
					Renal biopsy – Calcium oxalate crystals in tubular lumen	
2		58 F	45g IV×1 dose	Nephrotic syndrome	Oliguria. Treated with dopamine and hemodialysis. After first dialysis plasma vitamin C 15.4mg/dl (0.87mM), oxalate 2.3mg/dl. Intractable ventricular fibrillation. Post mortem- intra tubular calcium oxalate crystals.	Died(22)
				Creatinine 0.8		
3		61 M	60g IV×1 dose	Metastatic prostate cancer	Anuric. Creatinine 13.4. Plasma vitamin C 116.2mg/dl (6.6mM). Treated with nephrostomy and forced diuresis. Renal biopsy - acute tubular necrosis and extensive oxalate deposition	Recovered(23)
				Obstructive uropathy		
				Creatinine 0.7		
4	Hemolysis in Patients with Glucose-6-Phosphate Dehydrogenase Deficiency	68 M	80g IV×2 days	Second degree burns of one hand	Hemoglobin 5.8. Retics 5.9%. Anuria, creatinine 13.8. Coma, hemiparesis, possible intravascular coagulation. Supportive treatment and hemodialysis.	Died on day 22(25)
5		32 M	40g IV 3×/wk 20–40g/day oral×1 month then 80g IV×1 dose	HIV	Breathlessness, fever, dark urine. Hemoglobin 6.7 Retics 15.6%. Bilirubin 3.16. Conservative treatment with high fluid intake	Recovered(26)

Normal ranges and units of measurement for laboratory values are: Serum creatinine - mg/dl (normal range 0.6–1.5mg/dl). Hemoglobin g/dl (normal range: male 13–18g/dl, female 12–16g/dl). Reticulocyte count in % (normal range 0.5–2.5% red cells). Plasma bilirubin- mg/dl (normal range <1mg/dl). Plasma vitamin C - mg/dl (normal range 0.6–2 mg/dl).

### Practitioner Demographics

86% of practitioners were physicians (Table 6) and most patients were treated at for-profit centers. Some practitioners did not provide requested demographics data. Therefore, the numbers given in this table do not tally with the total number of survey respondents. (For detailed demographic information, see Table S4).

**Table 6:** Details of treating practitioners and institutions.

		Number of Practitioners	
		2006	2008
**Characteristics of Practitioners**	**Physician (MD)**	66	49
	**Doctor of Osteopathy (DO)**	11	12
	**Doctor of Naturopathy (ND)**	8	8
	**Nurse**	2	1
	**Physician's Assistant (PA)**	2	2
**Type of Institution**	**For-Profit organization**	81	68
	**Non-Profit/Other** *	7	4

*Other includes an academic medical center and a tribal medical center.

## Discussion

The data here show that 11,233 and 8876 patients received IV vitamin C over two periods of one year each, with a mean number of infusions per patient of 19–24 and a mean dose of approximately 28 grams per patient. We estimate that survey respondents used approximately 318,539 and 354,647 dosing units of vitamin C each year. These numbers account for less than half of the doses of vitamin C doses sold within the United States for the matching year. Considered together, these data indicate that use of IV vitamin C was both substantial and probably underestimated. This is one of the first papers to document previously unrecognized and widespread use of a CAM agent administered IV. To our knowledge, only two other CAM therapies are used IV. The first, chelation therapy, is also used in standard medical practice [27]. The second, an IV vitamin and mineral mixture termed the Myer's cocktail, has variable components, has had little formal investigation, and contains less than 5 grams of vitamin C [28], [29]. Further, there have been few surveys of CAM practitioners, as opposed to surveys of patients. There were minimal adverse effects reported, which was also the case in the published literature. Exceptions were for patients with pre-existing renal insufficiency/failure or glucose 6-phosphate dehydrogenase (G6PD) deficiency, both known to predispose to vitamin C toxicity [30]. Adverse events reported to the FDA could not be interpreted due to confounding factors.

Soon after its discovery and synthesis in 1932, parenteral vitamin C was shown to significantly decrease polio virus infections in primates [31], [32]. Although these findings were not repeatable [33], [34], one practitioner treated thousands of patients with parenteral vitamin C, primarily for infections, and popularized its use [2], [3], [5]. Such reports probably were a basis for continued use of parenteral vitamin C by other CAM practitioners [6], [7], [35]. Independently, others postulated that vitamin C could be useful in cancer treatment by enhancing or strengthening collagen and intercellular matrix synthesis and thereby decreasing metastases [36], [37]. Ewan Cameron, joined by Nobelist Linus Pauling, reported in retrospective case series that oral and IV vitamin C might benefit patients with advanced cancers [9], [10]. Placebo-controlled double blinded clinical trials at the Mayo Clinic showed no efficacy [38]–[40] but CAM practitioners continued to use IV vitamin C [11], [13], [35], consistent with our survey results. Pharmacokinetics evidence [15], [41], [42] now reveals that the exclusively oral vitamin C doses used in the Mayo studies would have produced peak plasma concentrations of approximately 0.2 mM, while the same dose given IV would produce peak plasma concentrations approximately 25 fold higher [15]. Pharmacologic doses of vitamin C given IV may produce drug effects in many body tissues, mediated by hydrogen peroxide formation in extracellular fluid but not blood [18]–[20]. Emerging clinical data are consistent with plausibility [43] and safety [16], but whether there is benefit or harm in humans can only be addressed by rigorous clinical trials.

IV vitamin C administered in gram doses can cause serious side effects in some patients. A metabolic end-product of vitamin C metabolism is oxalate, and oxalate nephropathy has been reported in patients with renal impairment given gram doses of IV vitamin C [22]–[24]. Prolonged treatment with vitamin C increases plasma oxalate concentrations in patients with renal failure [44] and results in increased urinary oxalate in patients receiving total parenteral nutrition [45], although in these patient groups the consequences of hyperoxalemia are unknown. Patients with glucose 6-phosphate dehydrogenase deficiency can develop intravascular hemolysis when gram doses of vitamin C are given IV [25], [26]. Vitamin C, even with oral dosing, might induce hemolysis in patients with Paroxysmal Nocturnal Hemoglobinuria [46], [47]. A recent phase one study of high dose IV vitamin C in patients with advanced cancer did not find any serious side effects [16]. Exclusion criteria for the phase I study included renal failure and glucose-6-phosphate dehydrogenase deficiency. Recently, a study in mice reported decreased efficacy of cancer chemotherapeutic agents with parenteral dehydroascorbic acid, a metabolite of vitamin C [48]. Dehydroascorbic acid cannot be detected in human blood or tissues, is not commercially available for parenteral use nor used by CAM practitioners, is toxic at high concentrations [49], [50], and should not be administered parenterally in humans.

No definitive serious adverse events were reported by survey respondents. Despite the anonymity of the survey, practitioners may have been reluctant to describe adverse events. Whether IV vitamin C is safe for general use remains to be determined. Because of the possibility of unrecognized side effects or of drug interactions, practitioners should enquire whether their patients, especially those with chronic, intractable or difficult to treat conditions, are receiving high dose IV vitamin C treatment elsewhere. Physicians should be alert to potential interactions of high dose vitamin C not only with conventional medicines but also with CAM treatments. An example is the case of exacerbated, severe cyanide poisoning in a patient on concurrent treatment with high dose oral vitamin C and Amygdalin (laetrile, a metabolic product of which is cyanide) [51].

Our study has limitations that, when considered together, may underestimate use of IV vitamin C. Because our survey was distributed to participants in a CAM conference, the survey excluded the vast majority of CAM practitioners. Respondents who filled in the survey form did so from memory without access to records, so that the information obtained can only be considered approximate. The format of the survey questions may have inadvertently resulted in underestimating vitamin C use, because the survey questions used ranges, for simplicity. For example, the highest value listed for numbers of patients treated in a calendar year was “>40”. If practitioners did not specify precise numbers as requested in subsequent questions, 40 was used as the number of patients treated, although the true number may have been higher. That there was only 24% overlap between survey respondents in 2006 and 2008 provides additional evidence that community use of IV vitamin C was underestimated. Data concerning industry sales of IV ascorbate only give approximations of use. On one hand, because not all units sold would have been used, use may be overestimated. Conversely, other smaller companies and compounding pharmacies may supply parenteral vitamin C but were not included in the survey. Therefore, the total number of vials sold may also be underestimated. The exact number of doses of parenteral vitamin C used in the US per year remains unknown. The survey response rate of approx 35% suggests we underestimated use, and perhaps adverse events. A reduced response rate may have occurred because some conference attendees were not practitioners and therefore did not return survey forms. Because of these uncertainties, the number of US patients treated with IV vitamin C cannot be accurately estimated from survey data. Since a primary aim of the survey was to determine if IV administered vitamin C is in use, and not a census of patients treated, the response rate does not detract from the value of the data.

Data obtained on adverse effects from PUBMED and the FDA also have limitations. Physicians may have not reported complications because they were not recognized, or were delayed, or were not attributed to vitamin C. Most practitioners do not report or publish adverse events. There may be as yet unknown adverse effects or interactions of IV ascorbate with other drugs. Side effects reported to FDA are difficult to interpret because of confounding factors. All reported patients received other potentially toxic drugs and/or had other diagnoses that may have been responsible for the reported adverse effects (see Table S3). Because of the FDA adverse events format, the same patient was likely to have been reported multiple times. Because of the low doses of IV vitamin C in the FDA dataset, it is highly unlikely that any of the reported adverse effects were due to the vitamin. Despite this, and because the information available in the FDA database is limited, it is not possible to accurately determine whether vitamin C caused or contributed to the reported side effects.

IV vitamin C is already in wide use, and physicians should know that their patients may seek IV vitamin C treatment in addition to conventional therapies. Beneficial effects of intravenous vitamin C on the disease conditions for which it is used are unproven, but side effects appear to be minor. Physicians should be cognizant of potential adverse or other unexpected effects, and of unrecognized interactions with drugs used in conventional and alternative medicine. CAM practitioners have an obligation to screen patients and should not administer high dose IV vitamin C to patients with pre existing renal disease, renal insufficiency or renal failure; glucose 6-phosphate dehydrogenase deficiency; a history of oxalate nephrolithiasis; or paroxysmal nocturnal hemoglobinuria. Based on emerging evidence, vitamin C in pharmacologic concentrations appears to be a pro-drug for delivery of hydrogen peroxide to the extravascular space. High dose IV vitamin C appears to have a positive safety profile, favorable pharmacology, evidence for mechanism of action, some anti-cancer effects *in vitro* and in animals, and widespread use outside conventional medicine with minimal harm, but without any proven clinical benefit.

## Supporting Information

Table S1 (0.18 MB DOC)

Table S2 (0.05 MB DOC)

Table S3 (0.17 MB DOC)

Table S4 (0.04 MB DOC)

Survey S1 Survey Form
(0.05 MB DOC)

## References

[1] Nutrition Business Journal (2008). NBJ's Supplement Business Report 2008: An Analysis of Markets, Trends, Competition and Strategy in the U.S. Dietary Supplement Industry. Figure 3–33(Table 1); Figure 4–18.

[2] Klenner FR (1949). The treatment of poliomyelitis and other virus diseases with vitamin C.. South Med Surg.

[3] Klenner FR (1951). Massive doses of vitamin C and the virus diseases.. South Med Surg.

[4] Calleja HB, Brooks RH (1960). Acute hepatitis treated with high doses of vitamin C. Report of a case.. Ohio Med.

[5] Klenner FR (1971). Observations on the dose and administration of ascorbic acid when employed beyond the range of a vitamin in human pathology.. Journal of Applied Nutrition.

[6] Cathcart RF (1891). Vitamin C, titrating to bowel tolerance, anascorbemia, and acute induced scurvy.. Med Hypotheses.

[7] Riordan NH, Riordan HD, Meng X, Li Y, Jackson JA (1995). Intravenous ascorbate as a tumor cytotoxic chemotherapeutic agent.. Med Hypotheses.

[8] Pauling L (1976). Vitamin C the common cold and the flu.

[9] Cameron E, Pauling L (1976). Supplemental ascorbate in the supportive treatment of cancer: Prolongation of survival times in terminal human cancer.. Proc Natl Acad Sci U S A.

[10] Cameron E, Pauling L (1978). Supplemental ascorbate in the supportive treatment of cancer: reevaluation of prolongation of survival times in terminal human cancer.. Proc Natl Acad Sci U S A.

[11] Riordan NH, Riordan HD, Casciari JJ (2000). Clinical and experimental experiences with intravenous vitamin C.. J Orthomolecular Med.

[12] Levy TE (2002). Vitamin C, Infectious Diseases, and Toxins: Curing the Incurable.

[13] Gonzalez MJ, Miranda-Massari JR, Mora EM, Guzman A, Riordan NH, Riordan HD (2005). Orthomolecular oncology review: ascorbic acid and cancer 25 years later.. Integr Cancer Ther.

[14] Levine M, Espey MG, Chen Q (2009). Losing and finding a way at C: new promise for pharmacologic ascorbate in cancer treatment.. Free Radic Biol Med.

[15] Padayatty SJ, Sun H, Wang Y, Riordan HD, Hewitt SM, Katz A (2004). Vitamin C pharmacokinetics: implications for oral and intravenous use.. Ann Intern Med.

[16] Hoffer LJ, Levine M, Assouline S, Melnychuk D, Padayatty SJ, Rosadiuk K (2008). Phase I clinical trial of i.v. ascorbic acid in advanced malignancy.. Ann Oncol.

[17] Padayatty SJ, Levine M (2000). Reevaluation of ascorbate in cancer treatment: emerging evidence, open minds and serendipity.. J Am Coll Nutr.

[18] Chen Q, Espey MG, Krishna MC, Mitchell JB, Corpe CP, Buettner GR (2005). Pharmacologic ascorbic acid concentrations selectively kill cancer cells: action as a pro-drug to deliver hydrogen peroxide to tissues.. Proc Natl Acad Sci U S A.

[19] Chen Q, Espey MG, Sun AY, Lee JH, Krishna MC, Shacter E (2007). Ascorbate in pharmacologic concentrations selectively generates ascorbate radical and hydrogen peroxide in extracellular fluid in vivo.. Proc Natl Acad Sci U S A.

[20] Chen Q, Espey MG, Sun AY, Pooput C, Kirk KL, Krishna MC (2008). Pharmacologic doses of ascorbate act as a prooxidant and decrease growth of aggressive tumor xenografts in mice.. Proc Natl Acad Sci U S A.

[21] Verrax J, Calderon PB (2009). Pharmacologic concentrations of ascorbate are achieved by parenteral administration and exhibit antitumoral effects.. Free Radic Biol Med.

[22] Lawton JM, Conway LT, Crosson JT, Smith CL, Abraham PA (1985). Acute oxalate nephropathy after massive ascorbic acid administration.. Arch Intern Med.

[23] Wong K, Thomson C, Bailey RR, McDiarmid S, Gardner J (1994). Acute oxalate nephropathy after a massive intravenous dose of vitamin C.. Aust N Z J Med.

[24] McAllister CJ, Scowden EB, Dewberry FL, Richman A (1984). Renal failure secondary to massive infusion of vitamin C.. JAMA.

[25] Campbell GD, Steinberg MH, Bower JD (1975). Ascorbic acid-induced hemolysis in G-6-PD deficiency.. Ann Intern Med.

[26] Rees DC, Kelsey H, Richards JD (1993). Acute haemolysis induced by high dose ascorbic acid in glucose-6- phosphate dehydrogenase deficiency.. BMJ.

[27] Barnes PM, Powell-Griner E, McFann K, Nahin RL (2004). Complementary and alternative medicine use among adults: United States 2002.. Adv Data.

[28] Gaby AR (2002). Intravenous nutrient therapy: the “Myers' cocktail”.. Altern Med Rev.

[29] Ali A, Njike VY, Northrup V, Sabina AB, Williams AL, Liberti LS (2009). Intravenous micronutrient therapy (Myers' Cocktail) for fibromyalgia: a placebo-controlled pilot study.. J Altern Complement Med.

[30] Levine M, Rumsey SC, Daruwala R, Park JB, Wang Y (1999). Criteria and recommendations for vitamin C intake.. JAMA.

[31] Jungeblut CW (1935). Inactivation of poliomyelitis virus in vitro by crystalline vitamin C (ascorbic acid).. Journal of Experimental Medicine.

[32] Jungeblut CW (1937). Further observations on vitamin C therapy in experimental poliomyelitis.. Journal of Experimental Medicine.

[33] Sabin AB (1939). Vitamin C in relation to experimental poliomyelitis.. Journal of Experimental Medicine.

[34] Jungeblut CW (1939). A further contribution to vitamin C therapy in experimental poliomyelitis.. Journal of Experimental Medicine.

[35] Riordan HD, Jackson JA, Schultz M (1990). Case study: high-dose intravenous vitamin C in the treatment of a patient with adenocarcinoma of the kidney.. J Orthomolecular Med.

[36] McCormick WJ (1954). Cancer: the preconditioning factor in pathogenesis; a new etiologic approach.. Arch Pediatr.

[37] Cameron E, Rotman D (1972). Ascorbic acid, cell proliferation, and cancer.. Lancet.

[38] Moertel CG, Fleming TR, Creagan ET, Rubin J, O'Connell MJ, Ames MM (1985). High-dose vitamin C versus placebo in the treatment of patients with advanced cancer who have had no prior chemotherapy. A randomized double-blind comparison.. N Engl J Med.

[39] Creagan ET, Moertel CG, O'Fallon JR, Schutt AJ, O'Connell MJ, Rubin J (1979). Failure of high-dose vitamin C (ascorbic acid) therapy to benefit patients with advanced cancer. A controlled trial.. N Engl J Med.

[40] Wittes RE (1985). Vitamin C and cancer.. N Engl J Med.

[41] Levine M, Conry-Cantilena C, Wang Y, Welch RW, Washko PW, Dhariwal KR (1996). Vitamin C pharmacokinetics in healthy volunteers: evidence for a Recommended Dietary Allowance.. Proc Natl Acad Sci U S A.

[42] Levine M, Wang Y, Padayatty SJ, Morrow J (2001). A new recommended dietary allowance of vitamin C for healthy young women.. Proc Natl Acad Sci U S A.

[43] Padayatty SJ, Levine M (2006). Vitamins C and E and the prevention of preeclampsia.. N Engl J Med.

[44] Canavese C, Petrarulo M, Massarenti P, Berutti S, Fenoglio R, Pauletto D (2005). Long-term, low-dose, intravenous vitamin C leads to plasma calcium oxalate supersaturation in hemodialysis patients.. Am J Kidney Dis.

[45] Pena dl V, Lieske JC, Milliner D, Gonyea J, Kelly DG (2004). Urinary oxalate excretion increases in home parenteral nutrition patients on a higher intravenous ascorbic acid dose.. JPEN J Parenter Enteral Nutr.

[46] Iwamoto N, Kawaguchi T, Horikawa K, Nagakura S, Hidaka M, Kagimoto T (1994). Haemolysis induced by ascorbic acid in paroxysmal nocturnal haemoglobinuria.. Lancet.

[47] Iwamoto N, Nakakuma H, Ota N, Shimokado H, Takatsuki K (1994). Ascorbic acid-induced hemolysis of paroxysmal nocturnal hemoglobinuria erythrocytes.. Am J Hematol.

[48] Heaney ML, Gardner JR, Karasavvas N, Golde DW, Scheinberg DA, Smith EA (2008). Vitamin C antagonizes the cytotoxic effects of antineoplastic drugs.. Cancer Res.

[49] Patterson JW (1950). The diabetogenic effect of dehydroascorbic and dehydroisoascorbic acids.. J Biol Chem.

[50] Patterson JW (1951). Course of diabetes and development of cataracts after injecting dehydroascorbic acid and related substances.. Am J Physiol.

[51] Bromley J, Hughes BG, Leong DC, Buckley NA (2005). Life-threatening interaction between complementary medicines: cyanide toxicity following ingestion of amygdalin and vitamin C.. Ann Pharmacother.

