# Supplementary material for: Vitamin C: Intravenous Use by Complementary and Alternative Medicine Practitioners and Adverse Effects
Source: PLoS One. 2010 Jul 7;5(7):e11414. doi: 10.1371/journal.pone.0011414 (PMC2898816; doi:10.1371/journal.pone.0011414)
Supplement: Table S1 — (0.18 MB DOC) [file pone.0011414.s001.doc]

| Number of practitioners who indicated that they treated the following conditions | | | | | |
| --- | --- | --- | --- | --- | --- |
| **Indication for Treatment - Cancer** | **Number of practitioners** | **Indication for Treatment - Infection** | **Number of practitioners** | **Indication for Treatment - Other** | **Number of practitioners** |
| Breast | 26 | Lyme | 27 | Energy / Fatigue | 41 |
| Colon | 20 | Viral Infection | 27 | Fibromyalgia | 17 |
| Prostate | 17 | Flu | 25 | Chelation (Prevent Cardio Disease) | 10 |
| Lung | 16 | Upper Respiratory Infection | 17 | Heavy Metal Detox / Toxins | 13 |
| Lymphoma | 16 | Hepatitis C | 11 | Health Maintenance / Nutritional | 8 |
| Ovarian | 16 | Cold | 11 | Meyers Cocktail | 5 |
| Pancreatic | 6 | Hepatitis (unspecified) | 10 | Mercury Amalgam filling removal | 5 |
| Rectal | 5 | Epstein-Barr | 10 | Immune Support | 5 |
| Bladder | 4 | Herpes | 9 | Candida | 5 |
| Gastric | 4 | Mononucleosis | 8 | Malabsorbtion | 3 |
| Uterine | 4 | Pneumonia | 6 | Prophylaxis (not defined) | 3 |
| Cervical | 3 | Bacterial | 6 | Allergy | 2 |
| Kidney | 3 | Sinusitis | 5 | Mycoplasma | 2 |
| Melanoma | 3 | Strep | 4 | Coronary Artery Disease | 2 |
| Unknown Metastases | 3 | Staph | 3 | Peripheral Artery Disease | 2 |
| Squamous cell carcinoma of tonsil | 2 | Bronchitis | 3 | Weakness, Soreness | 2 |
| Brain | 2 | Cytomegalovirus | 2 | Heart Disease | 2 |
| Cholangiocarcinoma | 2 | Mycoplasma | 1 | Stomach Bypass (post surgery) | 2 |
| Liver | 2 | Rhinovirus | 1 | Migraine | 2 |
| Mesothelioma | 2 | Tonsilitis | 1 | Urinary Tract Infection | 2 |
| Sarcoma | 2 | HIV | 1 | Parkinson’s Disease | 2 |
| Chronic Lymphocytic Leukemia | 1 | Varicella | 1 | Cellulitis | 2 |
| Glioblastoma | 1 | Diarrhea | 1 | Macular Degeneration | 1 |
| Larynx | 1 | West Nile | 1 | Anaphylaxis | 1 |
| “Solid Tumor” | 1 | Systemic | 1 | Pain | 1 |
| Tongue | 1 | Pneomococcus | 1 | Fever | 1 |
|  |  | Brown Recluse Spider | 1 | Tissue Repair | 1 |
|  |  | AIDS | 1 | Chronic Obstructive Pulm. Dis. | 1 |
|  |  | GI Tract Infection | 1 | Atherosclerotic Cardiovascular Disease | 1 |
|  |  |  |  | Drug addictions | 1 |
|  |  |  |  | Colitis and Crohn's Disease | 1 |
|  |  |  |  | Atherosclerosis | 1 |
|  |  |  |  | Autistic Children | 1 |
|  |  |  |  | Neurological Disease | 1 |
|  |  |  |  | Insulin Resistance | 1 |
|  |  |  |  | Fungal | 1 |
|  |  |  |  | Anti Aging | 1 |
|  |  |  |  | Schizophrenia | 1 |
|  |  |  |  | Kidney Stone | 1 |
|  |  |  |  | Car Accident | 1 |
|  |  |  |  | Failure to Thrive | 1 |
|  |  |  |  | Ulcer | 1 |
|  |  |  |  | Depression | 1 |
|  |  |  |  | Hydrocephalus | 1 |
|  |  |  |  | Symporter defect | 1 |
|  |  |  |  | Psoriasis / Skin infections | 1 |
|  |  |  |  | Weight Change | 1 |
|  |  |  |  | Smokers | 1 |
|  |  |  |  | Diabetes | 1 |

Appendix Table 1A. Number of practitioners who treated the conditions listed (year 2006). Listed conditions are given as described by the practitioners.

| Number of practitioners who indicated that they treated the following conditions | | | | | |
| --- | --- | --- | --- | --- | --- |
| **Indication for Treatment - Cancer** | **Number of practitioners** | **Indication for Treatment - Infection** | **Number of practitioners** | **Indication for Treatment - Other** | **Number of practitioners** |
| Breast | 38 | Viral | 15 | Fatigue | 22 |
| Prostate | 27 | Hepatitis | 13 | Fibromyalgia | 9 |
| Lymphoma | 19 | Flu | 11 | Detox | 8 |
| Colon | 16 | URI | 11 | Autoimmune | 5 |
| Ovarian | 14 | Lyme | 10 | Immune Enchancement | 5 |
| Lung | 10 | Pneumonia | 7 | Chelation | 4 |
| Pancreatic | 10 | Epstein-Barr | 6 | Health Promotion | 4 |
| Squamous cell carcinoma | 5 | Cold | 5 | Allergy | 2 |
| Uterine | 5 | Mono | 4 | Cosmetic + Antiaging | 2 |
| Esophageal | 4 | Bacterial | 4 | Meyers Cocktail | 2 |
| “All types” | 4 | Herpes | 3 | CFIDs | 2 |
| Multiple Myeloma | 4 | Shingles | 3 | Malabsorption | 2 |
| Hepatocellular Carcinoma | 4 | HIV | 2 | Pregnancy Related | 2 |
| Stomach/Gastric | 4 | Sinus | 2 | Palliative | 2 |
| Brain | 4 | Cellulitis | 2 | Macular degeneration | 2 |
| Head and Neck | 3 | Candida | 2 | Infectious Dis. | 1 |
| Mesothelioma | 3 | Pneumococcus | 2 | Acne | 1 |
| Bone | 3 | Staph | 2 | PCOS | 1 |
| Rectal | 2 | Strep | 2 | Narcotic Withdrawal | 1 |
| Metastatic of unknown origin | 2 | Diarrhea | 1 | Detox General Anesthesia Post-Op | 1 |
| Skin | 2 | HP infection | 1 | Depression | 1 |
| Adeno of unknown origin | 2 | Intestinal Infection | 1 | Adrenal Fatigue Syndrome | 1 |
| Thyroid | 2 | Mycoplasma | 1 | Bronchitis | 1 |
| Glioblastoma | 2 | West Nile | 1 | Multiple Chemical Sensitivity | 1 |
| Leukemia | 2 | Chronic Unknown | 1 | Autism | 1 |
| Sarcoma | 2 | Skin | 1 | Celiac | 1 |
| Hepatoma | 1 | Fever | 1 | Crohns | 1 |
| Osteosarcoma | 1 | Fungi | 1 | Removal of dental amalgam | 1 |
| Others | 1 | Varicella | 2 | COPD | 1 |
| Vaginal | 1 | Bell's Palsy | 1 | Rheumatoid arthritis | 1 |
| "Metastatic" | 1 | Bronchitis | 1 | Migrane | 1 |
| Merkel Cell | 1 | UTI | 1 | Cardiovascular disease | 1 |
| Abdominal Unknown | 1 | MRSA | 1 | Asthma | 1 |
| Nasal pharyngioma | 1 | Sore throat | 1 | Post polio | 1 |
| Solid Tumor | 1 |  |  | Enhance collagen formation | 1 |
| Renal Cell | 1 |  |  | Poison ivy | 1 |
| Lymphodema | 1 |  |  | Psoriasis | 1 |
| GIST | 1 |  |  | Diabetes | 1 |

| Melanoma | 1 |  |  | Hypertension | 1 |
| --- | --- | --- | --- | --- | --- |
| GALT tumor | 1 |  |  | Part of other protocols | 1 |
| Apendictal | 1 |  |  | Malnutrition | 1 |
| Myeloproliferative | 1 |  |  |  |  |
| Basal | 1 |  |  |  |  |
| Cervical | 1 |  |  |  |  |
| Wegners granulomatosis | 1 |  |  |  |  |
| Bowel | 1 |  |  |  |  |

Appendix Table 1B. Number of practitioners who treated the conditions listed (year 2008). Listed conditions are given as described by the practitioner.
