# Supplementary material for: Vitamin C: Intravenous Use by Complementary and Alternative Medicine Practitioners and Adverse Effects
Source: PLoS One. 2010 Jul 7;5(7):e11414. doi: 10.1371/journal.pone.0011414 (PMC2898816; doi:10.1371/journal.pone.0011414)
Supplement: Table S2 — (0.05 MB DOC) [file pone.0011414.s002.doc]

|  | **Number of Practitioners** | |
| --- | --- | --- |
| **Complications** | **2006** | **2008** |
| Lethargy / Fatigue | 13 | 14 |
| Vein Irritation | 5 | 4 |
| Nausea / Vomiting | 3 | 6 |
| Phlebitis | 3 | 3 |
| Kidney Stone | 2 | 2 |
| Hypoglycemia | 2 | - |
| Allergy | 2 | - |
| Blood Glucose Up | 2 | - |
| Hemolysis | 2 | - |
| Pain / Ache | 1 | 1 |
| Dry Mouth | 1 | - |
| Local Thrombosis | 1 | - |
| Headaches | 1 | 3 |
| Change in Mental State | 1 | 3 |
| Muscle Cramps | 1 | - |
| Syncope | 1 | 1 |
| Hematuria | 1 | - |
| Flu like symptoms | 1 | - |
| Cellulitis | 1 | - |
| Minor Intolerance | 1 | - |
| Herxheimer Reaction respond to Lyme | 1 | - |
| “Initiation of mem occasionally” | - | 1 |
| Venosclerosis | - | 1 |
| Mild palpitation | - | 1 |
| Renal Insufficiency or Renal Failure | - | 1 |
| Heartburn | - | 1 |
| Cold | - | 1 |
| Dizzyness | - | 1 |
| Other | - | 1 |

Appendix Table 2. All adverse effects reported by the survey respondents.

Side effects are listed as they were described by the survey respondents.
