# Supplementary material for: Vitamin C: Intravenous Use by Complementary and Alternative Medicine Practitioners and Adverse Effects
Source: PLoS One. 2010 Jul 7;5(7):e11414. doi: 10.1371/journal.pone.0011414 (PMC2898816; doi:10.1371/journal.pone.0011414)
Supplement: Table S3 — (0.17 MB DOC) [file pone.0011414.s003.doc]

Appendix Table 3. Adverse effects reported to the Food and Drug Administration (FDA) in patients treated with intravenous vitamin C. We searched the Adverse Events Reporting System, a database of drug side effects maintained by the FDA. We queried quarterly data available from the first quarter of 2004 to the last quarter of 2008 (a total of 20 quarters) and extracted data on patients treated with intravenous vitamin C. Most patients had severe and life threatening conditions and were on treatment with several drugs, many of which are known to have serious side effects. The dose of vitamin C administered was very low (1 gram or less) and it is unlikely that any of the reported adverse effects are attributable to intravenous vitamin C treatment. Note that we did not identify individual patients and the same patient may appear in several quarters, inflating the number of reported adverse events. Where the dose is given in ml, it is not possible to accurately determine the actual dose given. IV vitamin C preparations are usually supplied in 50 ml units containing 25g of Vitamin C. Therefore, when a dose of 0.5 ml is given, the administered dose is most likely 0.25g of vitamin C. Concurrent medications and side effects are reported exactly as in the FDA database.

| # | **Case number** | **Dose of vitamin C (IV)** | **Diagnosis or Indication for treatment** | **Concurrent Medication** | **Side Effects Reported** |
| --- | --- | --- | --- | --- | --- |
| 1 | 4323171 | 1G 5 times/week | Metastatic Malignant Melanoma | Arsenic Triseriox | Difficulty in Walking  Disease Progression  Dyspnoea  Metastasis  Nervous System Disorder  Pulmonary Vascular Disorder |
| 2 | 4330733 | 1G 5 times/week, followed by 1G twice/week | Metastatic Malignant Melanoma | Arsenic Triseriox | Disease Progression  Grand Mal Convulsion  Nervous System Disorder |
| 3 | 4336341 | 1G 5 times/week, followed by 1G twice/week | Metastatic Malignant Melanoma | Arsenic Triseriox  Temozolomide | Ascites  Confusional State  Dehydration  Delirium  Disease Progression  Dyspnoea  Lethargy  Mental Status Changes  Metastases to Central Nervous System  Progression of Neoplasm  Renal Failure  Somnolence |
| 4 | 4338117 | 1G 5 times/week, followed by 1G twice/week | Metastatic Malignant Melanoma | Arsenic Triseriox  Temozolomide | Abdominal Distension  Ascites  Confusional State  Dehydration  Delirium  Dyspnoea  Lethargy  Progression of Malignant Neoplasm  Metastases to Central Nervous System  Metastatic Malignant Melanoma  Brain Abnormal on Nuclear Magnetic Resonance Imaging  Renal Failure  Somnolence |
| 5 | 4343501 | 1G 5 times/week, followed by 1G twice/week | Metastatic Malignant Melanoma | Arsenic Triseriox  Temozolomide | Confusional State  Convulsion  Localized Oedema  Progression of Malignant Neoplasm  Nausea  Speech Disorder |
| 6 | 4348991 | Not Given | Not Given | Arsenic Triseriox  Temozolomide | Asthenia  Dyspnoea  Fatigue  Progression of Malignant Neoplasm  Metastases to Liver  Metastases to Lung  Metastases to Nervous System  Metastases to Retroperitoneum  Nausea  Pneumonia  Rash  Vomiting |
| 7 | 4349839 | 1G 5 times/week, followed by 1G twice/week | Metastatic Malignant Melanoma | Trisenox | Brain Oedema  Disease Progression  Grand Mal Convulsion  Nervous System Disorder |
| 8 | 4355682 | 1G 5 times/week, followed by 1G twice/week | Metastatic Malignant Melanoma  Malignant Neoplasm Progression  Metastases to Central Nervous System | Trisenox  Temozolomide | Confusional state  Convulsion  Progression of Malignant Neoplasm  Metastases to Central Nervous System  Nausea  Speech Disorder  Swelling |
| 9 | 4359361 | 1G 5 times/week, followed by 1G twice/week | Metastatic Malignant Melanoma | Trisenox  Temozolomide | Disease Progression  Grand Mal Convulsion  Nervous System Disorder |
| 10 | 4370904 | 1G Daily | Multiple Myeloma | Trisenox  Melphalan  Magnesium  Potassium  Zebeta  Lorazepam  Premarin | Acute Respirator Distress Syndrome  Cardiac Arrest  Congestive Cardiac Failure  Dyspnoea  Prolonged QT Interval on EKG  Loss of Consciousness  Ventricular Tachycardia |
| 11 | 4406594 | 1G Daily | Streptococcal Sepsis  Alcoholism | Amoxicillin  Clarithromycin  Pabrinex  Zinc  Folic Acid  Metoclopramide  Erythromycin  Tazocin  Ranitdine | Sepsis  Toxic Epidermal Necrolysis |
| 12 | 4507570 | 500 MG | Multiple Myeloma | Trisenox  Dexamethasone  Klacid (Clarithromycin Lactobionate)  Pamidronate Disodium | Dyspnoea  Fluid Retention |
| 13 | 4527828 | 1G | Multiple Myeloma | Trisenox  Zometa  Vitamin E  Calcium  Magnesium  Ibuprofen | Pyrexia  Acute Renal Failure  Sepsis |
| 14 | 4527839 | 1G 5 times/week | Metastatic Malignant Melanoma | Trisenox  Temozolomide | Ascites  Dehydration  Disease Progression  Progression of Malignant Neoplasm  Renal Failure  Somnolence |
| 15 | 4527840 | 1G 5 times/week | Metastatic Malignant Melanoma | Trisenox  Temozolomide | Increased Alanine Aminotransferase  Fatigue  Vomiting |
| 16 | 4565505 | Not given | Multiple Myeloma | Melphalan  Arsenic Trioxide  Enteric Aspirin  Nexium  K-Dur 10  Multi-vitamin  Iron Supplement  Aranesp  Zometa | T Wave Inversion on EKG |
| 17 | 4592645 | 0.5 mL daily | Insulin-dependent Diabetes Mellitus  Neonatal Asphyxia  Urinary Retention  Prophylaxis  Hypoxia | **Novolog**  Protaphan Penfill  Oxygen  Nikethamide  Menadione Sodium Bisulfite  Dipyrone INJ  Dimedrol  Relanium  Cefotaxime  Ethamsylate  Phenobarbital Tab  Dexmethsone  Magnesium Sulfate  Calcium Gluconate  Dopamine  Nystatin  Glucose  Panangin | **Central Nervous System Lesion**  Drug Exposure During Pregnancy  Neonatal Hypoclycaemia  **Ischaemia**  Neonatal Asphyxia  **Neonatal Hypoxia**  Premature Baby |
| 18 | 4605205 | 0.5 mL daily | Diabetes Mellitus Insulin-dependent  Neonatal Asphyxia  Urinary Retention  Prophylaxis  Hypoxia | Novorapid Penfill  Protaphan Penfill  Oxygen  Nikethamide  Menadione Sodium Bisulfite  Dipyrone INJ  Dimedrol  Relanium  Cefotaxime  Ethamsylate  Phenobarbital  Dexmethsone  Magnesium Sulfate  Calcium Gluconate  Dopamine  Nystatin  Glucose  Panangin  **Pregnavite**  **Vitamins NOS** | Maternal Drugs Affecting Foetus  Neonatal Hypoclycaemia  Neonatal Asphyxia  Premature Baby |
| 19 | 4635431 | 1G twice weekly | Multiple Myeloma | Trisenox  Melphalan | Progression of Malignant Neoplasm  Multiple Myeloma  Osteolysis |
| 20 | 4664057 | 1G twice weekly | Multiple Myeloma | Trisenox  Thalomid  Dexamethasone | Abdominal Pain  Dyspepsia  Perforation of Large Intestine |
| 21 | 4678599 | 0.5 mL daily | Diabetes Mellitus Insulin-dependent  Neonatal Asphyxia  Urinary Retention  Prophylaxis  Hypoxia | Novorapid Penfill  Protaphan Penfill  Oxygen  Nikethamide  Menadione Sodium Bisulfite  Dipyrone INJ  Dimedrol  Relanium  Cefotaxime Sodium  Ethamsylate  Phenobarbital  Dexmethsone  Magnesium Sulfate  Calcium Gluconate  Dopamine  Nystatin  Glucose  Panangin  Pregnavite  Vitamins NOS  Sulfacetamide Sodium | Central Nervous System Lesion  Drug Exposure During Pregnancy  Neonatal Hypoclycaemia  Neonatal Asphyxia  **Neonatal Disorder**  **Urinary Retention** |
| 22 | 4706607 | Not Given | Not Given | Tamiflu  PL  Medicon  Ambroxol Hydrochloride  Flomax  Soldem  Unspecified Other Medication | Haemorrhagic Enterocolitis  Escherichia Infection  Decreased Platelet Count |
| 23 | 4898762 | 1G daily | Multiple Myeloma | Velcade  Trisenox  Lisinopril  Plavix  Aspirin  Vicodin  Vitamin NOS | Pneumothorax  Pleural Effusion |
| 24 | 4910560 | Not Given | Acute Tonsillitis | Rocephin  Glucose  Aspara K  Diainamix | Decreased Blood Pressure  Drug Hypersensitivity  Increased Heart Rate  Urticaria |
| 25 | 4938215 | Not Given | Prophylaxis against Gstrointestinal Ulcer  Status Epilepticus  Parkinsonism  Pneumonia | Pepcid RPD  Pepcid  Valpronate Sodium  Benserazide Hydrochloride and Levodopa  Cercine  Dopaston  Cabaser  Antimicrobial (unspecified)  Cyanocobalamin  **Pyridoxine**  Riboflavin  Thiamine  Dextrose  Acetic Acid  Electrolytes (unspecified)  Amino Acids (unspecified) and Carbohydrates (unspecified) | **Convulsion**  Granulocytopenia  Decreased Platelet Count  Pneumonia  Sepsis  **Status Epilepticus** |
| 26 | 4951842 | Not Given | Prophylaxis against Gastrointestinal Ulcer  Status Epilepticus  Parkinsonism  Pneumonia | **Pyridoxine HCL**  Pepcid RPD  Pepcid  Valpronate Sodium  Benserazide Hydrochloride and Levodopa  Cercine  Dopaston  Cabaser  Antimicrobial (unspecified)  Cyanocobalamin  Riboflavin  Thiamine  Dextrose  Acetic Acid  Electrolytes (unspecified)  Amino Acids (unspecified) and Carbohydrates (unspecified) | Sepsis  Pneumonia  Decreased Platelet Count  Granulocytopenia |
| 27 | 4991701 | Not Given | Herpes Zoster  Parkinson’s Disease  Hypertension | Valitrex  Menesit  Nauzelin  Cabaser  Coniel  Diovan  Levodopa  Aspara K  Vitamin Supplement | Asthenia  Decreased Blood Chlorine  Increased Blood Creatine  Decreased Blood Potassium  Increased Blood Urea  Decreased Activity  Depressed Level of Consciousness  Acute Renal Failure  Somnolence |
| 28 | 5004379 | Not Given | Hyperlipidaemia | Zocor  Enalapril Maleate Amlodipine Hydrocloride  Brotizolam  Omeprazole,  Calcium polystyrene sulfonate  Dexamethasone Electrolytes and sodium lactate  Famotidine  NaCl  Vtamedin  Sulbactam sodium | Asthenia  Increased Blood Creatine Phosphokinase  Cholangitis |
| 29 | 5103427 | 1 G | Not Given | Alkeran  Arsenic Trioxide | Prolongation of Corrected QT Interval on EKG |
| 30 | 5123480 | Not Given | Pyrexia  Inflammation  Acute Bronchitis  Prophylaxis against Diarrhoea | Clarithromycin  Acetaminophen  Ibuprofen  PL  Levofloxacin  Lactobacillus Sporogenes  20% Glucose  Neolamin 3B INJ  Solita T3 Number 3 | Increased Alanine Aminotransferase  Arthralgia  Increased Aspartate Aminotransferase  Increased Bilirubin Conjugated  Increased Blood Alkaline Phosphatase  Increased Blood Bilirubin  Cholestasis  Increased Cough Gamma-Glutamyltransferase  Malaise  Productive Cough  Rash  Vomiting |
| 31 | 5148766 | 0.2 DF, QW3  0.2 daily followed by 2 times/week | Increased Blood Aluminum | Lactulose  Pantozol  Simvastatin  NPH Insulin  Methohexal  Tramadolor  Ferrlecit  Cyanocobalamin  Cinacalcet  Neorecormon  Calcitriol  Fesferal  Seroquel  Adumbran  Renagel  Axura | Increased Gamma-Glutamyltransferase |
| 32 | 5162818 | Not Given | Small Cell Lung Cancer Stage Unspecified | Hycamtin  Concurrent Medications | Alveolitis  Abonormal Blood Gases  Bronchiectasis  Death  Emphysema  Infection  Lung Consolidation  Pleural Fibrosis  Pulmonary Fibrosis  Pulmonary Haemorrhage  Mass Pulmonary Hilum  Pulmonary Oedema  Respiratory Failure |
| 33 | 5164724 | Not Given | Small Cell Lung Cancer Stage Unspecified | Hycamtin  Concurrent Medications | Alveolitis  Abonormal Blood Gases  Bronchiectasis  Death  Emphysema  Infection  Lung Consolidation  Lung Infection  Pleural Fibrosis  Pulmonary Haemorrhage  Mass Pulmonary Hilum  Pulmonary Oedema  Respiratory Failure  Recurrent Cancer |
| 34 | 5245516 | Not Given | Renal Cell Carcinoma Stage Unspecified | Su-011,248  Prevacid  Terazosin HCL  Effexor XR  Bisoprolol  Fumarate  Flaxseed Oil  Diflucan  Reactine  Oxeze Turbuhaler | Cerebrovascular Accident  Increased Lipase |
| 35 | 5267223 | Not Given | Hypocalcaemia | None | Anaphylactic Shock |
| 36 | 5275966 | Not Given | Hypocalcaemia | Rocalitrol  Calcicol  Fesin  Caltan  Mucosta  Myslee  Alfarol  Takepron  Fluitide Diskus  Serevent  Medicon  Codeine Phosphate | Anaphylactic Shock |
| 37 | 5276298 | Not Given | Pharyngitis | Zithromac  Loxonin  Transamin  Predonine  Gaster  Alfarol  Actonel | Diarrhoea  Reduced Fluid Intake  Generalized Erythema  Haemolytic Uraemic Syndrome  Inflammation  Acute Renal Failure  Skin Exfoliation  Viral Infection  Vomiting |
| 38 | 5304360 | Not Given | Hypocalcaemia | Rocalitrol  Calcicol  Fesin  Caltan  Mucosta  Myslee  Alfarol  Takepron  Fluitide Diskus  Serevent  Medicon  Codeine Phosphate | Anaphylactic Shock |
| 39 | 5308734 | Not Given | Hypocalcaemia | Rocalitrol  Calcicol  Fesin  Caltan  Mucosta  Myslee  Alfarol  Takepron  Fluitide Diskus  Serevent  Medicon  Codeine Phosphate | Anaphylactic Shock |
| 40 | 5311194 | Not Given | Rectal Cancer | Sodium Chloride 0.9% in Plastic Container  Furosemide | Blood Pressure  Chills  Pyrexia |
| 41 | 5317111 | Not Given | Hypocalcaemia | Rocalitrol  Calcicol  Fesin  Caltan  Mucosta  Myslee  Alfarol  Takepron  Fluitide Diskus  Serevent  Medicon  Codeine Phosphate | Anaphylactic Shock |
| 42 | 5322206 | Not Given | Hypocalcaemia | Rocalitrol  Calcicol  Fesin  Caltan  Mucosta  Myslee  Alfarol  Takepron  Fluitide Diskus  Serevent  Medicon  Codeine Phosphate | Anaphylactic Shock |
| 43 | 5356111 | Not Given | Pharyngitis | Zithromac  Loxonin  Transamin  Predonine  Gaster  Alfarol  Actonel | Generalized Erythema  Haemolytic Uraemic Syndrome  Acute Renal Failure |
| 44 | 5502167 | Bolus | Pneumonia  Gastric Ulcer Haemorrhage | Meropenem  Lac-Tac  Vitamedin  Lansoprazole  Adona  Transamine  Bisolvon | Abnormal Hepatic Function |
| 45 | 5618243 | Not Given | Cholecystitis | Tazocin  Electrolyte Solutions  Vitamins NOS  Riboflavin Tab  Thiamine Disulfide  Pyridoxine Hydrochloride  Vitamin B-12  Soldem 3  Amlodipine Besylate  Diovan  Nizatidine  Aspirin  Mobic  Aleviatin  Magnesium Oxide  Artist  Amino Acids Nos | Pneumonia |
| 46 | 5627413 | Not Given | Cholecystitsis  Hypertension  Myocardial Infarction  Subdural Haemorrhage | Tazocin  Electrolyte Solutions  Vitamins NOS  Riboflavin Tab  Thiamine Disulfide  Pyridoxine Hydrochloride  Vitamin B-12  Soldem 3  Amlodipine Besylate  Diovan  Nizatidine  Aspirin  Mobic  Aleviatin  Magnesium Oxide  Artist  Calonal  Piperacillin Sodium  Amino Acids Nos | Increase in Percentage of Eosinophils  Interstitial Lung Disease |
| 47 | 5663394 | Not Given | Pharyngitis | Cefzon  Acetaminophnen  PL Grand.  Veen D  Thiamine HCL  Flavine Adenine Dinucleotide | Anorexia  Increased Body Temperature  Dehydration  Liver Disorder  Oral Herpes  Toxic Epidermal Necrolysis  Ulcerative Keratitis |
| 48 | 5671007 | Not Given | Pharyngitis | Cefzon  Paracetamol  PL Grand.  Veen D  Thiamine HCL  Flavine Adenine Dinucleotide | Anorexia  Increased Body Temperature  Dehydration  Liver Disorder  Oral Herpes  Toxic Epidermal Necrolysis  Ulcerative Keratitis |
| 49 | 5671966 | Not Given | Cholecystitsis  Fluid Replacement  Nutritional Support  Hypertension  Myocardial Infarction  Subdural Haemorrhage  Constipation | Tazocin  Electrolyte Solutions  Vitamins NOS  Riboflavin Tab  Thiamine Disulfide  Pyridoxine Hydrochlorine  Vitamin B-12  Amlodin  Diovan  Nizatidine  Aspirin  Aleviatin  Magensium Oxide  Artist  Calona  Piperacillin Sodium  Amino Acids NOS | Increase in Percentage of Eosinophil  Interstitial Lung Disease |
| 50 | 5709319 | Not Given | Cholecystitis  Fluid Replacement  Nutritional Support  Hypertension  Myocardial Infarction  Subdural Haemorrhage  Constipation  Hypertension | Tazocin  Electrolyte Solutions  Vitamins NOS  Riboflavin Tab  Thiamine Difulfide  Pyridoxine Hydrochloride  Vitamin B12  Amlodin  Diovan  Nizatidine  Aspirin  Aleviatin  Magnesium Oxide  Artist  Calonal  Piperacillin Sodium  Amino Acids NOS | Eosinophil Percentage Increased  Interstitial Lung Disease  Cholecystitis  Fluid Replacement |
| 51 | 5710235 | Not Given | Pharyngitis | Cefzon  Paracetamol  PL Gran.  Veen D  Thiamine HCL  Flavine Adenine Dinucleotide | Anorexia  Body Temperature Increase  Dehydration  Liver Disorder  Oral Herpes  Toxic Epidermal Necrolysis  Ulcerative Keratitis |
| 52 | 5710306 | Not Given | Sedation  Fluid Replacement  Vomiting | Atarax  Solulact  Elieten  Solita-T3 Injection  Loxonin  Mucosta  Adofeed  Solita T  Thiamine HCL  Riboflavin Sodium Phosphate  Hipyridoxin  Panthenol | Generalized Erythema |

| 53 | 5721238 | Not Given | Pharyngitis | Cefzon  Paracetamol  PL Gran.  Veen D  Thiamine HCL  Flavine Adenine Dinucleotide | Anorexia  Body Temperature Increase  Dehydration  Liver Disorder  Oral Herpes  Toxic Epidermal Necrolysis  Ulcerative Keratitis |
| --- | --- | --- | --- | --- | --- |
| 54 | 5738960 | Not Given | Pharyngitis | Cefzon  Paracetamol  PL Gran.  Veen D  Thiamine HCL  Flavine Adenine Dinucleotide | Anorexia  Body Temperature Increase  Dehydration  Liver Disorder  Oral Herpes  Toxic Epidermal Necrolysis  Ulcerative Keratitis |

| 55 | 5739388 | Not Given | Sedation  Fluid Replacement  Vomiting  Pain | Atarax  Solulact  Elieten  Loxonin  Adofeed  Solita-T3 Injection  Mucosta  Solita T  Thiamine HCL  Riboflavin Sodium Phosphate  Hipyridoxin  Panthenol | Generalized Erythema |
| --- | --- | --- | --- | --- | --- |
| 56 | 5744372 | 1 D/F, DAILY (1/D) | Diabetes Mellitus | Humulin R  Vitamedin  Famotidine  Wystal  Hirudoid  Alesion  Alloid G  Excelase | Alanine Aminotransferase Increased  Aspartate Aminotransferase Increased |

| 57 | 5757003 | 1 D/F, DAILY (1/D) | Diabetes Mellitus | Humulin R  Vitamedin  Famotidine  Wystal  Hirudoid  Alesion  Alloid G  Excelase | Hepatic Function Abnormal |
| --- | --- | --- | --- | --- | --- |
| 58 | 5757548 | Not Given | Sedation  Fluid Replacement  Vomiting  Pain  Vitamin Supplementation | Atarax  Solulact  Elieten  Loxonin  Adofeed  Solita-T3 Injection  Mucosta  Solita T  Thiamine HCL  Riboflavin Sodium Phosphate  Hipyridoxin  Cyanocobalamin  Panthenol | Generalized Erythema |

| 59 | 5761457 | Not Given | Pharyngitis | Cefzon  Paracetamol  PL Gran.  Veen D  Thiamine HCL  Flavine Adenine Dinucleotide | Anorexia  Body Temperature Increase  Dehydration  Liver Disorder  Oral Herpes  Toxic Epidermal Necrolysis  Ulcerative Keratitis |
| --- | --- | --- | --- | --- | --- |
| 60 | 5772447 | 1 D/F, DAILY (1/D) | Diabetes Mellitus | Humulin R  Vitamedin  Famotidine  Wystal  Hirudoid  Alesion  Alloid G  Excelase | Hepatic Function Abnormal |

| 61 | 5774967 | Not Given | Dialysis  Hyperparathyroidism Secondary | Epogen  Sensipar  Doxercalciferol  Levocarnitine  Dapsone  Docusate Sodium  Fluconazole  Furosemide  Lanthanum Carbonate  Mycophenolate Mofetil  Pantoprazole Sodium  Simvastatin  Tacrolimus  Synthroid  Diltiazem  Metoprolol  Allegra  Flonase  Nephor-caps  Prinivil | Diarrhoea  Folate Deficiency  Kidney Transplant Rejection  Pyelonephritis  Therapeutic Response Decreased  Urinary Tract Infection |
| --- | --- | --- | --- | --- | --- |

| 62 | 5805503 | Not Given | Dialysis  Hyperparathyroidism Secondary | Epogen  Sensipar  Doxercalciferol  Levocarnitine  Dapsone  Docusate Sodium  Fluconazole  Furosemide  Lanthanum Carbonate  Mycophenolate Mofetil  Pantoprazole Sodium  Simvastatin  Tacrolimus  Synthroid  Diltiazem HCL  Metoprolol Succinate  Allegra  Flonase  Nephor-caps  Prinivil | Diarrhoea  Folate Deficiency  Kidney Transplant Rejection  Pyelonephritis  Therapeutic Response Decreased  Urinary Tract Infection |
| --- | --- | --- | --- | --- | --- |

| 63 | 5806265 | Not Given | Anaesthesia Procedure  Sedation  Antibiotic Therapy  Diuretic Therapy  Blood Pressure Management  Muscle Relaxant Therapy | Sevoflurane  Propofol  Insulin  Midazolam HCL  Ketamine Hydrochloride  Famotidine  Cefazolin Sodium  Teicoplanin  Vancomycin Hydrochloride  Ceftazidime  Vitamin B Complex Cap  Panthenol  Calcium Gloconate  Potassium Chloride  Furosemide  Potassium Canrenoate  Bifidobacterium  Nifedipine  Polymyxin B Sulfate  Vecuronium Bromide  Atropine Sulfate  Neostigmine Metilsulfate  Nitruous Oxide w/ Oxygen | Toxic Epidermal Necrolysis |
| --- | --- | --- | --- | --- | --- |
| 64 | 5811523 | Not Given | Peptic Ulcer  Vitamin Supplementation  Sedation  Antibiotic Level  Antibiotic Therapy  Polyuria  Hypertension  Anaesthesia  Pneumonia  Thermal Burn  Muscle Relaxant Therapy | Pepcid  Insulin Human  Ketamine Hydrochloride  Fentanyl Citrate  Teicoplanin  Vancomycin Hydrochloride  Ceftazidime  Cyanocobalamin and Pyridoxine and Thiamine  Panthenol  Calcium Gloconate  Potassium Chloride  Furosemide  Canrenoate Potassium  Bifidobacterium Animalis and Lactobacillus Acidophilus  Nifedipine  Polymyxin B Sulfate  Vecuronium Bromide  Propofol  Atropine Sulfate  Neostigmine Bromine  Nitrous Oxide  Sevoflurane  Nicardipine Hydrochloride  Cefazolin Sodium Midazolam | Toxic Epidermal Necrolysis |
| 65 | 5816089 | Not Given | Anaesthesia Procedure  Sedation  Blood Pressure Management  Antibiotic Therapy  Diuretic Therapy  Muscle Relaxant Therapy | Sevoflurane  Propofol  Insulin  Midazolam HCL  Ketamine Hydrochloride  Fentanyl Citrate  Nicardipine Hydrochloride  Cefazolin Sodium  Teicoplanin  Vancomycin Hydrochloride  Ceftazidime  Vitamin B Complex Cap  Panthenol  Calcium Gloconate  Potassium Chloride  Furosemide  Potassium Canrenoate  Bifidobacterium  Nifedipine  Polymyxin B Sulfate  Vecuronium Bromide  Atropine Sulfate  Neostigmine Metilsulfate  Nitruous Oxide Famotidine | Toxic Epidermal Necrolysis |

| 66 | 5826308 | Not Given | Peptic Ulcer  Sedation  Vitamin Supplementation  Antibiotic Level  Antibiotic Therapy  Polyuria  Hypertension  Anaesthesia  Pneumonia  Thermal Burn  Muscle Relaxant Therapy | Pepcid  Insulin Human  Ketamine Hydrochloride  Fentanyl Citrate  Teicoplanin  Vancomycin Hydrochloride  Ceftazidime  Cyanocobalamin and Pyridoxine and Thiamine  Panthenol  Calcium Gloconate  Potassium Chloride  Furosemide  Canrenoate Potassium  Bifidobacterium Animalis and Lactobacillus Acidophilus  Nifedipine  Polymyxin B Sulfate  Vecuronium Bromide  Propofol  Atropine Sulfate  Neostigmine Bromine  Nitrous Oxide  Sevoflurane  Nicardipine Hydrochloride  Cefazolin Sodium Midazolam  Vitamin B Complex  Propofol  Physio 35  Solita T-3  Ensure  Albumin Human | Toxic Epidermal Necrolysis |
| --- | --- | --- | --- | --- | --- |
| 67 | 5829165 | Not Given | Anaesthesia Procedure  Sedation  Blood Pressure Management  Antibiotic Therapy  Diuretic Therapy  Muscle Relaxant Therapy | Sevoflurane  Propofol  Insulin  Midazolam HCL  Ketamine Hydrochloride  Fentanyl Citrate  Nicardipine Hydrochloride  Cefazolin Sodium  Teicoplanin  Vancomycin Hydrochloride  Ceftazidime  Vitamin B Complex Cap  Panthenol  Calcium Gloconate  Potassium Chloride  Furosemide  Potassium Canrenoate  Bifidobacterium  Nifedipine  Polymyxin B Sulfate  Vecuronium Bromide  Atropine Sulfate  Neostigmine Metilsulfate  Nitruous Oxide Famotidine | Toxic Epidermal Necrolysis |

| 68 | 5840331 | Not Given | Peptic Ulcer  Sedation  Vitamin Supplementation  Antibiotic Level  Antibiotic Therapy  Polyuria  Hypertension  Anaesthesia  Pneumonia  Thermal Burn  Muscle Relaxant Therapy | Pepcid  Insulin Human  Ketamine Hydrochloride  Fentanyl Citrate  Teicoplanin  Vancomycin Hydrochloride  Ceftazidime  Cyanocobalamin and Pyridoxine and Thiamine  Panthenol  Calcium Gloconate  Potassium Chloride  Furosemide  Canrenoate Potassium  Bifidobacterium Animalis and Lactobacillus Acidophilus  Nifedipine  Polymyxin B Sulfate  Vecuronium Bromide  Propofol  Atropine Sulfate  Neostigmine Bromine  Nitrous Oxide  Sevoflurane  Nicardipine Hydrochloride  Cefazolin Sodium Midazolam  Vitamin B Complex  Propofol  Physio 35  Solita T-3  Ensure  Albumin Human | Toxic Epidermal Necrolysis |
| --- | --- | --- | --- | --- | --- |
| 69 | 5843048 | Not Given | Anaesthesia Procedure  Sedation  Blood Pressure Management  Antibiotic Therapy  Diuretic Therapy  Muscle Relaxant Therapy | Sevoflurane  Propofol  Insulin  Midazolam HCL  Ketamine Hydrochloride  Fentanyl Citrate  Nicardipine Hydrochloride  Cefazolin Sodium  Teicoplanin  Vancomycin Hydrochloride  Ceftazidime  Vitamin B Complex Cap  Panthenol  Calcium Gloconate  Potassium Chloride  Furosemide  Potassium Canrenoate  Bifidobacterium  Nifedipine  Polymyxin B Sulfate  Vecuronium Bromide  Atropine Sulfate  Neostigmine Metilsulfate  Nitruous Oxide Famotidine | Toxic Epidermal Necrolysis |

| 70 | 5845398 | Not Given | Peptic Ulcer  Sedation  Vitamin Supplementation  Antibiotic Level  Antibiotic Therapy  Polyuria  Hypertension  Anaesthesia  Pneumonia  Thermal Burn  Muscle Relaxant Therapy | Hydrochloride  Ceftazidime  Cyanocobalamin and Pyridoxine and Thiamine  Panthenol  Calcium Gloconate  Potassium Chloride  Furosemide  Canrenoate Potassium  Bifidobacterium Animalis and Lactobacillus Acidophilus  Nifedipine  Polymyxin B Sulfate  Vecuronium Bromide  Propofol  Atropine Sulfate  Neostigmine Bromine  Nitrous Oxide  Sevoflurane  Nicardipine Hydrochloride  Cefazolin Sodium Midazolam  Vitamin B Complex  Propofol  Physio 35  Solita T-3  Ensure  Albumin Human | Toxic Epidermal Necrolysis |
| --- | --- | --- | --- | --- | --- |
| 71 | 5874537 | 500mg | Ureteric Cancer | Zoledronic Acid  Kytril  Decadron SRC  Methotrexate  Exal 1  Neu-up  Oxycontin  Durotep Janssen  Novamin  Gasmotin  Maglax  Pursennid  Lecicarbon  Mobic  Rivotril  Loramet  Lactec  Lactec D  Fluid/Electrolyte Replacement Therapy  Tathion  Pantol  Fentanyl  Soldem 3A  Metabolin  Fladd  Lasix | Blood Urea Abnormal  Malignant Neoplasm Progression |
| 72 | 5908267 | Not Given | Diarrhoea  Large Intestine Carcinoma  Pain  Gastrointestinal Mucosal Disorder  Nausea  Fluid Replacement  Gastrointestinal Motility Disorder  Vitamin Supplementation  Blood Glucose Decreased  Nutritional Support  Prophylaxis | Lopemin  Irinotecan HCL  Tegafur/Gimeracil/Oteracil Potassium  Oxycotin  Buscopan  Mucosta  Voltaren  Novamin  Soldem 3A  Primperan Tab  Vitamedin Intravenous  Sodium Chloride  Glucose  Intravenous Nutrients  Nasea | Gastric Ileus  Lower Gastrointestinal Harmorrhage  Sepsis |
| 73 | 5926207 | 5g x 1 dose | Vitamin Supplementation  Mineral Supplementation | Calcium Gloconate  Magnesium Sulfate  Zinc  Chromium  Selenium | Diarrhoea  Infusion Related Reaction  Nausea  Vomiting  White Blood Cell Count Decreased |
| 74 | 5937093 | Not Given | Non-Small Cell Lung Cancer | Carboplatin  Taxol  Dexamethasone  Diphenhydramine HCL  Ondansetron  Ranitidine | Respiratory Failure |

| 75 | 5941369 | 100 PG | Cough  Productive Cough  Muscle Fibrosis | Itraconazole  Midazolam HCL  Medicon  Mucosolvan  Cotrim  Alfarol  Meropen  Minocycline HCL  Alinamin  Solu-medrol  Predonine  Famtodine  Bfluid | Disease Progression  Hepatic Function Abnormal  Pneumothorax |
| --- | --- | --- | --- | --- | --- |
| 76 | 5946044 | Not Given | Chemotherapy | Neulasta  Fluorouracil  Epirubicin  Cyclophosphamide | Cerebrovascular Accident  White Blood Cell Count Increased |
| 77 | 5975638 | 1 G Qwkly Days 1, 8, 15, And 22 Intravenous | Myelodysplastic Syndrome | Arsenic Trioxide  Decitabine | Febrile Neutropenia |
