# Supplementary material for: Vitamin C: Intravenous Use by Complementary and Alternative Medicine Practitioners and Adverse Effects
Source: PLoS One. 2010 Jul 7;5(7):e11414. doi: 10.1371/journal.pone.0011414 (PMC2898816; doi:10.1371/journal.pone.0011414)
Supplement: Table S4 — (0.04 MB DOC) [file pone.0011414.s004.doc]

|  |  | **Characteristics of Institution** | | | **Demographics** | | | |
| --- | --- | --- | --- | --- | --- | --- | --- | --- |
| **2006** | **Practitioner’s Degree** | **Profit** | **Non-profit** | **Other** | **Male** | **Female** | **Average Year of Graduation** | **Average Age** |
|  | Physician (MD) | 60 | 3 | 2 | 50 | 16 | 1979 | 54 |
|  | Doctor of Osteopathy (DO) | 11 | 0 | 0 | 10 | 1 | 1982 | 53 |
|  | Doctor of Naturopathy (ND) | 6 | 2 | 0 | 3 | 5 | 1993 | 48 |
|  | Nurse | 2 | 0 | 0 | 0 | 2 | 1978 | 55 |
|  | Physicians Assistant (PA) | 2 | 0 | 0 | 0 | 2 | 2000 | 43 |
| **2008** | Physician (MD) | 46 | 2 | 1 | 37 | 12 | 1982 | 54 |
|  | Doctor of Osteopathy (DO) | 11 | 1 | 0 | 8 | 4 | 1983 | 55 |
|  | Doctor of Naturopathy (ND) | 8 | 0 | 0 | 0 | 8 | 2004 | 35 |
|  | Nurse | 1 | 0 | 0 | 0 | 1 | 1981 | 60 |
|  | Physicians Assistant (PA) | 2 | 0 | 0 | 1 | 1 | 2000 | 55 |

Appendix Table 4. Characteristics of survey respondents. The majority of practitioners who used high dose IV vitamin C were physicians and they worked in for profit organizations. “Other” in 2006 includes an academic medical center and a tribal medical center. For 2008, “Other” was undefined.
