# Supplementary material for: Vitamin C: Intravenous Use by Complementary and Alternative Medicine Practitioners and Adverse Effects
Source: PLoS One. 2010 Jul 7;5(7):e11414. doi: 10.1371/journal.pone.0011414 (PMC2898816; doi:10.1371/journal.pone.0011414)
Supplement: Survey S1 — Survey Form (0.05 MB DOC) [file pone.0011414.s005.doc]

# Survey on Intravenous Vitamin C

The purpose of this 10-minute survey is to learn from physicians and health professionals about clinical experiences with intravenous vitamin C. These questions apply to your experiences and usage of intravenous vitamin C in the past 12 months. We request that you recall your experiences to the best of your ability. If you need more room, please use the other side of the page and number your response. This survey is completely anonymous and there are no identifiers. *If you responded to this survey in 2006, please respond again about your usage in the past 12 months, and also check here. _______*

**1. In the past 12 months, about how many patients have you treated with intravenous vitamin C in your practice? *Please circle one of the answers below.***

0 patients 1-10 patients 11-20 patients 21-40 patients >40 patients

If you can estimate the number more precisely, please do so here. __________patients

If you did not give any intravenous vitamin C in the past 12 months, please stop here, and thanks.

**Vitamin C Dose**

**2. What was the AVERAGE dose of intravenous vitamin C that you gave to patients, in grams per day. (For example: if you gave 15 grams/day on average, you should circle 11-20grams/day below). *Please circle one of the answers below.***

1-10grams/day 11-20grams/day 21-40grams/day 41-80grams/day >80grams/day

If you can precisely estimate the AVERAGE dose in grams/day you gave to patients in the past 12 months, please do so here. ________grams/day

**3. What was the LOWEST dose in grams per day of intravenous vitamin C that you gave patients in the past 12 months? ___________ grams/day**

**4. What was the HIGHEST dose in grams per day of intravenous vitamin C that you gave patients in the past 12 months? ___________grams/day**

**Infusion Rate and Duration of Infusion**

**5. What was the LOWEST infusion rate of intravenous vitamin C that you gave in the past 12 months? (Example, 0.2 grams per minute) *Please write this below.***

____________grams per minute

**6. What was the AVERAGE infusion rate and duration of infusion that you used in patients in the past 12 months? (Example – 1 gram per minute for 60 minutes.) *Please write this below.***

***____***_____________________

**7. What was the HIGHEST infusion rate of intravenous vitamin C that you gave in the past 12 months? (for example, 2 grams per minute). *Please write this below.***

____________grams per minute

**8. What was the SHORTEST total time of infusion of intravenous vitamin C, in minutes? (For example, 60 minutes) *Please write this below*.**

____________minutes

**9. What was the LONGEST total time of infusion of intravenous vitamin C, in minutes? (For example, 500 minutes) *Please write this below*.**

____________minutes

**Number of Doses**

**10. What was the AVERAGE number of intravenous doses that you gave per patient?**

1-10 doses 11-20 doses 21-40 doses 41-80 doses >80 doses

If you can estimate the AVERAGE number of doses given per patient more precisely, please do so here. _______ doses

**11**. **How many times per week or per month did you give intravenous vitamin C to patients, on average? *Please circle one of the answers below.***

Once weekly Twice weekly Three times weekly Once monthly Twice monthly

If another frequency, please describe here. __________________

**12. What was the SMALLEST number of doses of intravenous vitamin C that you gave to a single patient in the past 12 months?**

1-10 doses 11-20 doses 21-40 doses 41-80 doses >80 doses

**13. What was the LARGEST number of doses of intravenous vitamin C that you gave to a single patient in the past 12 months?**

1-10 doses 11-20 doses 21-40 doses 41-80 doses >80 doses

**14. What is the total number of doses of intravenous vitamin C that you gave to all patients in the past 12 months? To answer this, please add up all of the doses that you gave to all of your patients in the past 12 months. *Please circle one choice below.***

1-10 11-20 21-50 51-100 101-200 >200

If you can estimate the TOTAL numbers of doses given to ALL patients in the past one year more precisely, please do so here. ________total number of doses

**Indications**

**15. Why did you give intravenous vitamin C? (Check all that apply, and then provide the additional details).**

**___Cancer Treatment**

How many patients did you treat for cancer using intravenous vitamin C in the past 12 months? ______patients

Please specify the number and types of cancers

**____Treatment of Infection,**

How many patients did you treat for infection with intravenous vitamin C in the past 12 months? ___________patients

Please specify suspected infectious agents

**____Other Reasons**

Number of patients treated for Other reasons in the past 12 months ______patients

Please specify the reasons for treatment

**Complications or Adverse Events**

16. In the past 12 months, did any of your patients experience any complications or adverse events while treated with intravenous vitamin C?

_____No

_____Yes.

If yes, please indicate below which of the complications or adverse events were observed, and please indicate how many times each complication or adverse event occurred, in the past 12 months.

_____Kidney Stones

_____Hemolysis

_____Renal Insufficiency or Renal Failure

_____Lethargy/Fatigue

_____Change in Mental Status

_____Other

17. If your patients had any complications in the past 12 months, please tell us what else the patients had concurrently received and provide details, if possible.

# Additional Information and Opinions

**18. If as part of your practice you have administered intravenous vitamin C for more than one year: for approximately how many years have you been administering intravenous vitamin C? *Please write your answer below:***

***__________________*years**

19. What else would you like us to know about your use of intravenous vitamin C in your practice?

The remaining questions are included so that we will know about the health care providers who completed this survey and the characteristics of their practices. Again, please note that this survey is completely anonymous.

**20. What is your gender?**

___Male

___Female

21. What is your medical/professional degree?

____MD

____DO

____ND

____NP

____PA

____Other

22. In what year did you graduate from medical, osteopathic, nursing, physician assistant, or other professional school?

_______

(year)

**23. What is your age? _____________**

**24. What is your type of practice?**

_____For Profit
_____Non Profit

_____Foundation

_____Other – please describe

**25. Did you respond to this survey in 2006?**

**_____ Yes**

**_____ No**

**Thank you for participating.**
